# Factors Influencing Range of Motion after Total Knee Arthroplasty

**Published:** 2012-07-30

**Authors:** H Farahini, M Moghtadaei, A Bagheri, E Akbarian

**Affiliations:** 1 Department of Orthopedics Surgery, Rasule-Akram General Hospital, Tehran University of Medical Sciences, Tehran, Iran; 2 Department of Public Health, Karolinska Institute, Campus Solna, Sweden

**Keywords:** Arthroplasty, Motion, Flexion, Knee

## Abstract

**Background:**

The range of motion after a total knee arthroplasty is an important clinical outcome affecting the life of the patient. The aim of this study was to determine the most important factors influencing the postoperative knee flexion in Tehran, Iran.

**Methods:**

Between July 2007 and January 2009, on 95 cases of total knee joint replacement (89 patients), who were followed for 1 year postoperatively, the risk factors were assessed. Patient demographics (sex, age, body mass index, previous surgery, preoperative Knee Society System score, and preoperative range of motion) as well as radiographic measurement for preoperative tibiofemoral angle were statistically analyzed and the probable predictors entered into a linear regression model.

**Results:**

Univariate analysis showed that age, preoperative flexion angle, preoperative flexion arc and preoperative tibiofemoral angle had significant correlation with the postoperative flexion angle. The linear regression model on the other hand revealed that preoperative flexion angle and preoperative tibiofemoral angle were the true predictors of the postoperative flexion angle with coefficients of 0.64 and -0.21, respectively.

**Conclusion:**

Better range of motion before the surgery with a lower tibiofemoral varus/valgus angle were more likely to result in a better range of motion after the surgery, suggesting that an appropriate timing for the surgery when the knee joint is still in a better function can lead to a better outcome.

## Introduction

The increased need for an effective procedure for painful arthritic disorders, remaining physically active, strong marketing by the implant companies, and better patient education have led to joint replacement surgeries among younger ages than 60 years old patients.[1] Two main measures of a successful total knee arthroplasty are increase in the range of motion and relief from pain.[2][3][4][5][6] Range of motion is one of the most important criteria for the patient to be satisfied with the surgery and is directly related to the patient’s being physically active.[1][2] Satisfactory flexion is needed for various daily activities: 67º for swing phase of gait, 83º for climbing up the stairs, 90º for descending stairs, and 93º for standing up from a chair.[7][8] Some studies have reported that limited range of motion before the surgery considerably improved after total knee arthroplasty, while knees with a good preoperative range of motion stayed the same or had reduced range of motion after the surgery.[2][6] On the other hand, there were some studies stating that the limited preoperative range of motion was likely to result in limited postoperative range of motion.[9][10] In addition, patients with preoperative moderate or severe flexion contractures showed improvement after total knee replacement.[10][11] Several risk factor assessment studies mentioned preoperative range of motion, tibiofemoral varus/valgus angle, underlying disease, age and weight (in relation with height) of the patient, preoperative physical activity level of the patient, surgical technique, implant design, and postoperative physiotherapy as factors which have impact on the range of motion postoperatively.[2][3][5][6][9][12][13][14] Other influencing factors in the postoperative range of motion comprise the height of postoperative joint line, patellar diameter, and preoperative level of pain.[15] The aim of the present study was to assess influencing factors on the postoperative range of motion of the knee among the patients who underwent total knee arthroplasty in Rasoule-Akram General Hospital, Tehran, Iran.

## Materials and Methods

This study was a retrospective risk factor assessment on 95 cases of total knee joint replacement (89 patients), who consecutively underwent the arthroplasty procedure between July 2007 and January 2009 in Rasoule-Akram General Hospital, Tehran, Iran. The patients who had a systemic underlying disease or were candidates of revision total knee arthroplasty were excluded from the study. Followed by an informed consent for the study, demographic information of the patients along with history of medical situation including osteoarthritis, rheumatoid arthritis, or previous surgical procedures on the knee were recorded. Moreover, iron supplement was prescribed for 4 weeks before the surgery and non-steroid anti inflammatory drugs (NSAIDs) were discontinued one week before the arthroplasty.

Total knee joint replacement was performed either under general or local anesthesia by or under direct supervision of the first author. In all cases, the same surgical technique by means of cemented implants was used and the cement was enriched by gentamycin antibiotic. Pneumatic tourniquet was used for all the patients to stop blood flow during the surgery, while suction drain was applied after the surgery. After finishing the procedure, the knee was dressed carefully as well as supported by the knee brace, so that it was fixed in the extension. Heparin as a prophylactic anticoagulant agent was administered right before the surgery and was substituted by warfarin for the next 4 weeks. Passive movements and weight bearing were started in all patients 2 days after the surgery, when the drain was taken out.

Postoperative follow-up scheduled between 2 to 4 weeks after the surgery and patients were checked for hematoma or other operative consequences. All patients underwent clinical and radiographic examinations before the surgery as well as the third month and the first year after the arthroplasty, while the range of motion which obtained before the surgery and after one year were used for the evaluation of the outcome. Range of motion of the knee, including flexion and extension, was achieved by means of a goniometer. The tibiofemoral angle was measured by simple radiographs and the Knee Society Clinical Rating System Score (KSS) was also assessed as the protoco l[16] to provide with a functional evaluation.

Demographic, clinical, and radiologic data of the patients including age, sex, body mass index (BMI), preoperative diagnosis, range of motion, tibiofemoral angle, and KSS were collected and analyzed with STATA software for windows (STATA, Version 10, TX, USA) using Chi-Square and Wilcoxon signed-ranked tests, paired samples t and Pearson correlation tests. In addition, linear regression model was carried out to identify the probable influencing factors on the postoperative range of motion of the knee. In the current study, the level of significance has been defined to be less than 0.05.

## Results

Among 89 patients with the mean age of 65±6.3 years, ranging from 58 to 79 years old, 38 individuals (42.7%) were male and 51 (57.3%) were female; whereas, 23 patients (25.8%) were under and 66 ones (74.2%) were above 65 years old. Mean weight of patients was 71.3±7.8 years, ranging from 61 to 90 kilograms, and mean height was 1.6±0.06 cm, ranging from 1.53 to 1.74 meters (Table 1). 45 cases (47.4%) underwent total joint arthroplasty of the right knee, while 50 ones (52.6%) had this procedure on the left side. The underlying disease was osteoarthritis in 85 patients and rheumatoid arthritis in only 3 individuals. There was a history of tibial osteotomy in 8 patients, knee arthroscopy in 21, and meniscectomy in 10 individuals.

**Table 1:** Mean weight of patients.

**General information**		**No (%)**
Age (years)	65±6.3 (58 – 79)	
	< 65	23 (25.8)
	> 65	66 (74.2)
Gender	Male	38 (42.7)
	Female	51 (57.3)
Weight (kilograms)	71.3±7.8 (61 – 90)	
Height (meters)	1.6±0.06 (1.53 – 1.74)	
Knee side	Right	45 (47.4)
	Left	50 (52.6)
Underlying disease	Osteoarthritis	85
	Rheumatoid arthritis	3
Surgical history	Tibial osteotomy	8
	Arthroscopy	21
	Meniscectomy	10

The average length of time for the surgery was 88.1±17.4 minutes, ranging from 70 to 135 min, and the amount of bleeding during the operation was averagely 196.1±87 milliliters, ranging from 50 to 400 ml. Mean admitted days in the hospital was 6.1±1.5 days, ranging from 4 to 11 days, for the patients. No surgical consequences like nerve paralysis, delayed healing wound, or deep vein thrombosis was seen; however, there were 2 cases of surgical site infection and 3 cases of knee hematoma after the surgery.

As shown in Table 2, preoperative range of motion among the patients comprised mean flexion of 101.6±14.3º, mean flexion arc of 97.9±31.3º, and mean extension of 5.7±4.8º; whereas, mean postoperative values were 106.3±11.1º for flexion, 100.2±25.6º for flexion arc, and 1.4±3.1º for extension. There were significant differences between all the average measures for the range of motion before and after the arthroplasty, where the difference in mean flexions was 4.7º (p-value=0.001), flexion arcs of 2.3º (p-value=0.03), and extensions -4.3 (p-value=0.001). Additionally, the KSS score, which evaluated the clinical situation and function of the joint, was 45.2±12.1 before the surgery, significantly increased to 93.7±10.8 after the operation (p-value=0.001). Studying patients’ radiographs revealed that the mean of tibiofemoral angle was 181.6±11.4º before the arthroplasty, which was remarkably decreased to 176.6±3.3º postoperatively (p-value=0.001).

**Table 2:** Pre- and postoperative information.

	**Preoperative**	**Postoperative**	***P* value**
Flexion	101.6±14.3º	106.3±11.1º	< 0.001
Flexion arc	97.9±31.3º	100.2±25.6º	0.03
Extension	5.7±4.8º	1.4±3.1º	< 0.001
TF angle^a^	181.6±11.4º	176.6±3.3º	< 0.001
KSS score^b^	45.2±12.1	93.7±10.8	< 0.001

^a^ Tibiofemoral angle

^b^ Knee Society System score

Pearson correlation coefficient was calculated between the postoperative flexion range and age, BMI, flexion, flexion arc, extension, tibiofemoral angle, and KSS score of the patients, preoperatively. Results of the analysis showed that age (r=-0.102, p-value=0.04), preoperative flexion (r=0.365, p-value=0.001), preoperative flexion arc (r=0.316, p-value=0.003), and tibiofemoral angle (r=-0.285, p-value=0.007) had a significant correlation with the postoperative flexion. Moreover, using an independent samples t-test between the genders regarding postoperative flexion range showed no significant difference among the two genders. The recently related mentioned factors therefore entered in a linear regression model along with the postoperative flexion to define which one had significant influence on the results of flexion range after the surgery. The model could significantly (p-value of the model=0.001) predicted almost 28% of the variations (adjusted R-square=0.279) of the postoperative flexion angle, while preoperative values of flexion, tibiofemoral angle, flexion arc, and age are all in the model. As shown in Table 3, preoperative flexion angle (t=4.87, p-value=0.001) had the most positive effect on the postoperative flexion (β coefficient=0.64) followed by preoperative tibiofemoral angle (t=-2.6, β coefficient=-0.211, p-value=0.01); whereas, the other two factors of preoperative flexion arc and age had no significant effect on the postoperative flexion. According to the regression analysis, by increasing 10º in preoperative flexion angle, the postoperative flexion would rise by almost 6.5º, while by reducing 10º of preoperative tibiofemoral angle, the flexion would be increased by almost 2.1º postoperatively.

**Table 3:** Linear regression analysis for predicting postoperative flexion angle.

	***β*-coefficient**	**Standard error**	***t*-statistic**	***P *value**	**95% confidence interval**	
Preoperative flexion	0.643	0.132	4.87	<0.001	0.38	0.9
Preoperative TF angle^a^	-0.211	0.081	-2.6	0.01	-0.372	-0.05
Preoperative flexion arc	0.414	0.236	1.75	0.08	-0.055	0.883
Age	-0.035	0.041	-0.85	0.39	-0.116	0.046

^a^ Preoperative tibiofemoral angle, R-square=0.32, Adjusted R-square=0.279, P value<0.001

## Discussion

The knee joint arthroplasty is the standard treatment for severe dysfunction of the knee aiming to make the knee pain free as well as stabilize the knee with an appropriate range of motion. Progress in the knee implant design and the surgical techniques for total knee replacement achieved successful results in reducing the pain and providing with a stable joint; however, enhancing the postoperative range of motion is yet challengeable. The postoperative range of motion is one of the major criteria of the patient’s satisfaction of the arthroplasty, where the patient needs an acceptable flexion of the knee for many of daily activities.

As expected, the present study revealed that all the preoperative measures of the range of motion, including flexion, flexion arc, and extension significantly enhanced after the surgery. Moreover, the mean preoperative tibiofemoral varus/valgus angle, which was calculated from radiophotographs of the knee, decreased remarkably after the arthroplasty. Besides, the clinical evaluation of the patients, assessed by the KSS score significantly improved almost two times the preoperative values after the operation. This study followed the patients up for one year and the results of the above mentioned factors after one year of the surgery built up the postoperative values, which was a limitation for the study since long-term results might be different at least for some of the studied variables. On the other hand, the most effective clinical outcomes of the knee joint arthroplasty present themselves almost one year after the surgery,[17] which makes this study valid enough to assess the factors influencing postoperative range of motion.

Gender showed to have no significant relationship with the postoperative flexion, which was similar with most of the previous reports;[14][18][19] however, there was a few articles discussing it as a factor related to the final range of motion outcome.[20] In addition, there was no relationship between body mass index and the postoperative flexion values, which was compatible with some of the previous studies [18][19] and against some others,[21][22] which have found this relationship. We also found no correlation between history of the previous surgical procedures on the knee, including tibial osteotomy, knee arthroscopy, and meniscectomy with postoperative flexion, similar to the findings of Kawamura et al.[18] Although univariate analysis found a minor significant correlation between age and postoperative flexion (r=-0.102, small effect size), compatible with some previous studies,[20][23] the regression analysis revealed that age had no significant effect on the postoperative range of motion, which was compatible with most of the previous reports.[2][12][18][19] Furthermore, preoperative values for flexion (r=0.365, strong effect size), flexion arc (r=0.316, strong effect size), and tibiofemoral varus/valgus angle (r=-0.285, medium effect size) had significant correlations with postoperative flexion angle in their univariate analyses.

The linear regression model, which was further developed in our analysis, included four main factors having remarkable correlation with the postoperative flexion angle, including age and preoperative values for flexion, flexion arc, and tibiofemoral angle. This model could truly predict the postoperative flexion outcome, using the two variables of preoperative flexion and preoperative tibiofemoral varus/valgus angle in a way that by increasing 10º of preoperative flexion, the outcome flexion would likely to be enhanced by 6.4º, holding the other factors constant, and by decreasing 10º of preoperative tibiofemoral angle, the outcome flexion would likely to be improved by 2.1º, holding the other factors unchanged. Our findings were compatible with most of the previous publications regarding the influence of preoperative flexion degree on postoperative flexion outcome.[12][13][18][20][21][23] Likewise, many previous articles have reported the influence of preoperative tibiofemoral varus/valgus angle on the results of postoperative flexion;[9][10][13][18] nonetheless, some others suggested that there is no relation between the outcome and the preoperative tibiofemoral angle values.[19][20]

Based on the findings of this study, the two factors of preoperative flexion angle and preoperative tibiofemoral varus/valgus angle had a significant influence on the postoperative flexion, which means they were important factors for predicting the outcome range of motion of the knee after a total knee arthroplasty. Therefore, preoperative function of the knee had a major role on the outcome function, suggesting that a total knee arthroplasty is better to be carried out at an appropriate time, when the knee joint is still has a better function, without being so destructed by the underlying disease, mainly osteoarthritis.
